# Potential of an Isolated Bacteriophage to Inactivate *Klebsiella pneumoniae*: Preliminary Studies to Control Urinary Tract Infections

**DOI:** 10.3390/antibiotics13020195

**Published:** 2024-02-19

**Authors:** João Duarte, Carolina Máximo, Pedro Costa, Vanessa Oliveira, Newton C. M. Gomes, Jesús L. Romalde, Carla Pereira, Adelaide Almeida

**Affiliations:** 1 CESAM, Department of Biology, University of Aveiro, Campus Universitário de Santiago, 3810-193 Aveiro, Portugal; j.macedoduarte@ua.pt (J.D.); carolina.maximo@ua.pt (C.M.); pedrommrscosta@ua.pt (P.C.); v.oliveira@ua.pt (V.O.); gomesncm@ua.pt (N.C.M.G.); 2 Department of Microbiology and Parasitology, CRETUS & CIBUS, Faculty of Biology, University of Santiago de Compostela, CP 15782 Santiago de Compostela, Spain; jesus.romalde@usc.es

**Keywords:** bacteriophage, phage therapy, *Klebsiella pneumoniae*, urinary tract infections

## Abstract

Urinary tract infections (UTIs) caused by resistant *Klebsiella pneumoniae* can lead to severe clinical complications and even death. An alternative treatment option for infected patients is using bacteriophages. In the present study, we isolated phage VB_KPM_KP1LMA (KP1LMA) from sewage water using a *K. pneumoniae* strain as a host. Whole-genome analysis indicated that the genome was a double-stranded linear 176,096-bp long DNA molecule with 41.8% GC content and did not contain virulence or antibiotic resistance genes. The inactivation potential of phage KP1LMA was assessed in broth at an MOI of 1 and 10, and a maximum inactivation of 4.9 and 5.4 log CFU/mL, respectively, was observed after 9 h. The efficacy at an MOI of 10 was also assessed in urine to evaluate the phage’s performance in an acidic environment. A maximum inactivation of 3.8 log CFU/mL was observed after 9 h. The results suggest that phage KP1LMA could potentially control a UTI caused by this strain of *K. pneumoniae,* indicating that the same procedure can be used to control UTIs caused by other strains if new specific phages are isolated. Although phage KP1LMA has a narrow host range, in the future, efforts can be made to expand its spectrum of activity and also to combine this phage with others, potentially enabling its use against other *K. pneumoniae* strains involved in UTIs.

## 1. Introduction

The World Health Organization has classified ESKAPEE, a group of seven highly virulent bacteria (*Enterococcus faecium*, *Staphylococcus aureus*, *Klebsiella pneumoniae*, *Acinetobacter baumannii*, *Pseudomonas aeruginosa*, *Enterobacter* spp. and *Escherichia coli*), as number one priority for the development of new antimicrobial drugs [1]. Among these, the Gram-negative bacterium *K. pneumoniae* poses a significant global health threat due to the widespread dissemination of carbapenemase genes [2]. In clinical settings, *K. pneumoniae* is a common cause of bloodstream, respiratory, and urinary tract infections (UTIs) [3]. Notably, in intensive care units, most healthcare-associated infections are associated with endotracheal tubes (cases of pneumonia), vascular catheters (bloodstream infections), or urinary catheters (UTIs) [4]. According to the 2019 European Centre for Disease Prevention and Control (ECDC) report, 94% of UTIs were associated with the presence of urinary catheters [4]. The adhesive fimbriae of *K. pneumoniae* enables the formation of robust biofilms on medical devices [5]. These biofilms confer additional resistance against antibiotics, complicating clinical outcomes and limiting treatment options [6]. In the most recent antimicrobial resistance surveillance report, *K. pneumoniae* isolates presented widespread resistance to third-generation cephalosporins and increasing resistance to carbapenems in a worsening scenario across Europe [7]. The resistance to these antibiotics can also be transferred to other bacteria during the biofilm matrix’s formation, affecting the treatment options available for other bacteria [6]. Complications arise in patients with UTIs where antimicrobial-resistant bacteria are detected, leading to prolonged hospitalizations, higher rates of treatment failure, and even death [8]. As the antimicrobial treatment landscape worsens, there is an urgent need for novel treatment options in clinical settings.

Phages, which are viruses specific to bacteria, offer several advantages over antibiotics, including low environmental impact, preservation of the microbiota, ease and cost-effectiveness of isolation, and resistance to cross-resistance [9]. Furthermore, phages can infect antibiotic-resistant bacteria and, in some cases, enhance bacterial susceptibility to antibiotics where resistance has been observed [10]. Therefore, researchers worldwide are exploring their potential applications in various fields [11]. Given that patients in intensive care units are prone to UTIs [12,13], the use of phages to treat these infections has received considerable attention [14]. Commercially available phage preparations from the ELIAVA Institute have been used against isolated *E. coli* and *K. pneumoniae* strains obtained from UTI patients [15]. These phages were even directly administered to treat UTIs in patients who underwent transurethral resection of the prostate [16]. In a different approach, Le and his coworkers (2023) [17] successfully treated a recurrent UTI caused by *K. pneumoniae* through intravenous administration of phages without antibiotics. Other researchers have isolated and characterized phages specific to *K. pneumoniae*, testing their efficacy against biofilm formation and in animal models [18,19]. However, some isolated and characterized phages exhibit a very narrow host range and cannot infect different bacterial strains of the same species [9]. Implementing phage libraries may address this limitation, allowing researchers to select the most effective phage preparations for a specific strain [20]. As the levels of *K. pneumoniae* resistance to carbapenems continue to increase in Europe and specifically in Portugal [7], the isolation and characterization of new phages capable of infecting this bacterium have become crucial for providing new treatment options for patients. Therefore, this study focuses on isolating and characterizing a new phage capable of infecting an environmentally isolated carbapenemase-producing *K. pneumoniae* strain. In addition, in vitro tests in liquid medium and human urine were conducted to assess the phage’s potential for use in urinary tract infections, providing insight into its efficacy in the acidic environment of the urinary tract.

## 2. Results

### 2.1. Phage Isolation and Virion Morphology

**Figure 1:**
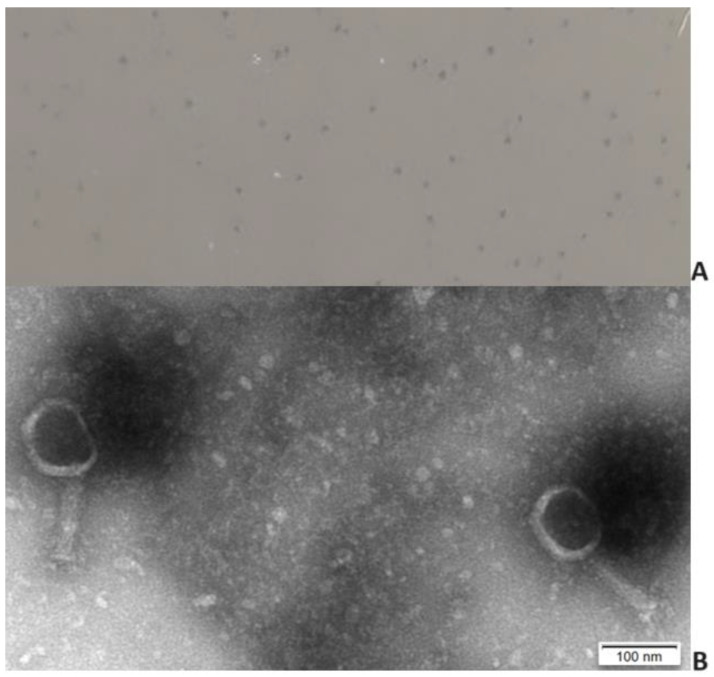
Phage plaque morphology (**A**) and electron micrograph photography (**B**).

Phage KP1LMA formed very small (diameter <0.1 mm) clear plaques on the host strain (Figure 1A). High-titer suspensions [10^9^ plaque-forming units (PFU/mL)] were produced. Transmission electron microscopy revealed virions with an icosahedral head of approximately 76 nm and a contractile tail of 110 ± 7.9 nm (Figure 1B). Phage KP1LMA presents a myovirus morphotype and has been classified as *Caudoviricetes*.

### 2.2. Genome Analysis

**Figure 2:**
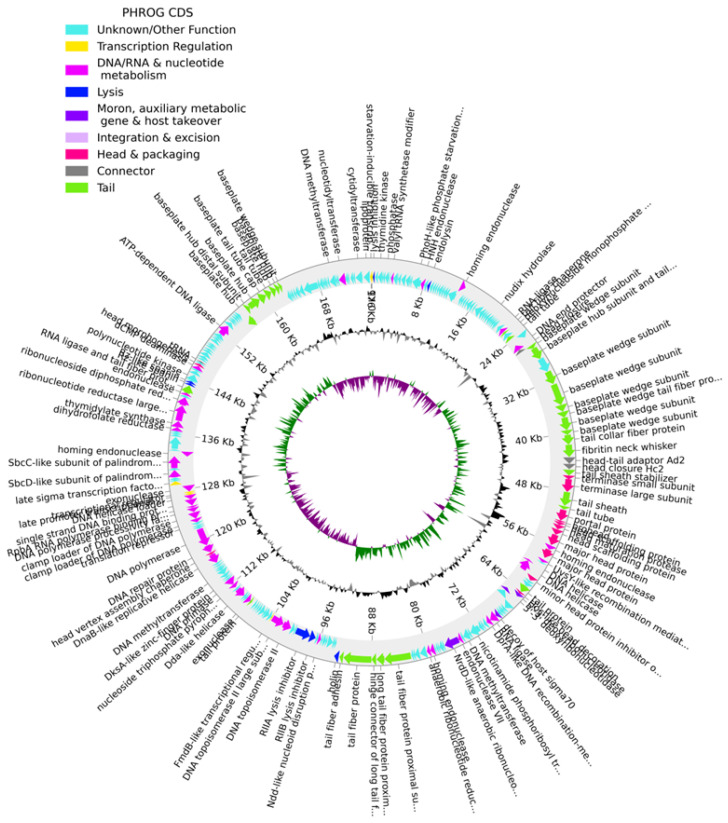
The genome map of phage KP1LMA. The outer circle with arrow-headed bands represents the coding DNA sequences (CDS) color coded according to the functional category of the predicted gene in the direction of the transcription. The innermost ring represents the genome GC skew (green/pink) followed by GC content (black/grey). The labels show the predicted functions of the functional CDSs, color-coded by the PHROGs category (Supplementary Table S1).

The genome of phage KP1LMA (GenBank: PP002985) is a double-stranded DNA with a size of 176,096 bp, and the GC content is 41.8%. The CheckV results showed 98.8% of completeness and no host contamination. A total of 268 CDSs were predicted in the phage KP1LMA genome, with 148 CDSs predicted as hypothetical proteins and 120 CDSs as functional proteins (Figure 2, Table S1). Our analysis could not detect genes encoding antibiotic resistance or virulence determinants in the phage KP1LMA genome. The absence of the phage integrase gene indicates that phage KP1LMA could potentially be exploited for biocontrol applications. We further detected the presence of two tRNA genes in the KP1LMA genome, suggesting that the phage may rely on its tRNA for translation after infecting the host cell (Figure 2, Table S1). Overall, we detected putative genes encoding for phage proteins involved in DNA replication and modification, phage structure, and packaging and host lysis (e.g., endolysin and holin) (Figure 2, Table S1). The genome sequence of phage KP1LMA was classified under the *Slopekvirus* genus and had a similarity of 98.87% with *Klebsiella* phage phiKp_26 (GenBank: LC768496) as well with a set of *Klebsiella* phages isolated from sewage. Comparative genome analysis of phage KP1LMA and phage phiKP_26 (GenBank: LC768496) revealed large similarity between genomes (Table S2).

### 2.3. Phage Host Range and Efficiency of Plating

The spot test analysis revealed that phage KP1LMA presented a very narrow host range infecting only *E. coli* ATCC 13706 of the 52 strains tested, with an efficiency of plating of 32.98 ± 2.85% (Table 1). Phages isolated from the plaques formed on the *E. coli* strain could still infect the *K. pneumoniae* strain used as the host.

### 2.4. Adsorption Curve

**Figure 3:**
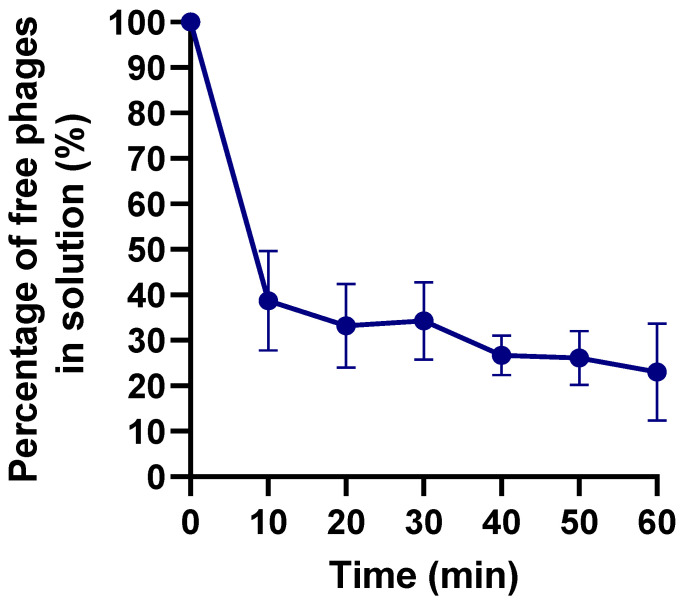
Adsorption curves of phages KP1LMA in the presence of *K. pneumoniae* as the host. Values represent the mean of three independent experiments, and error bars represent the standard deviation.

The KP1LMA phage adsorption assays showed that the phage adsorbed at a constant K = 4.23246 × 10^−10^ mL^−1^ min^−1^, and approximately 60% and 75% of the phage particles adsorbed to *K. pneumoniae* Scc 24 cells after 10 and 60 min, respectively (Figure 3). 

### 2.5. One-Step Growth Curve

**Figure 4:**
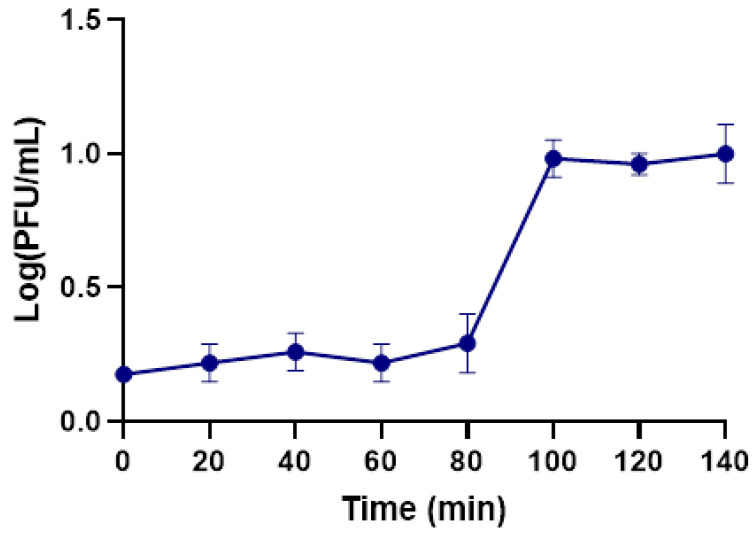
One-step growth curve of phage KP1LMA in the presence of the host, *K. pneumoniae*. Values represent the mean of three independent experiments, and error bars represent the standard deviation.

The growth curve for phage KP1LMA was determined in Tryptic Soy Broth (TSB, Liofilchem, Roseto degli Abruzzi, Italy) at 37 °C (Figure 4). From the observed results, phage KP1LMA presented a latent period of 100 min and a burst size of 7.9 ± 0.3 PFU/host cell.

### 2.6. Bacterial Kill Curve

**Figure 5:**
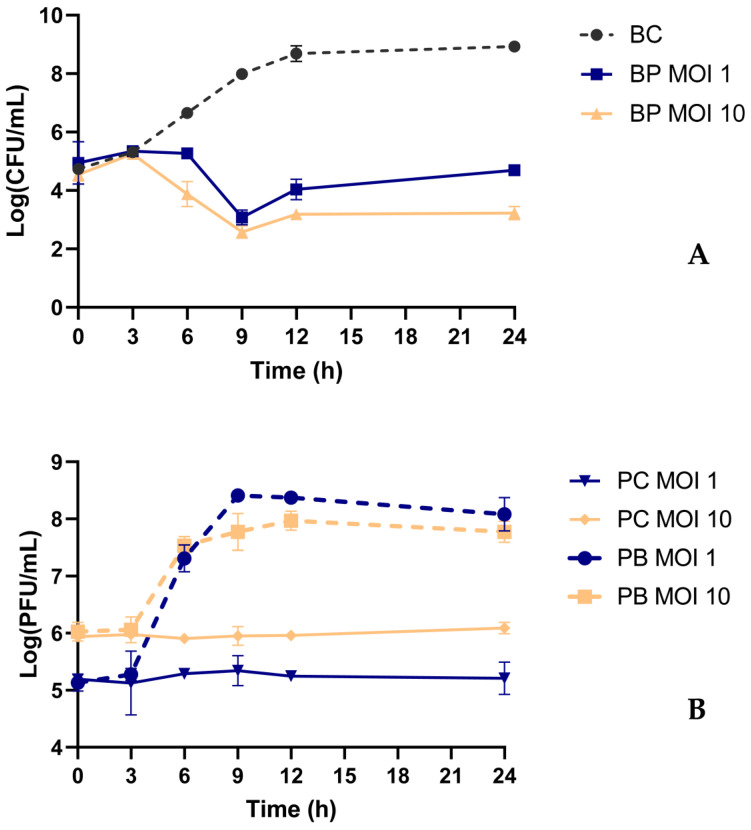
Inactivation of *K. pneumoniae* using phage KP1LMA at two different MOIs. (**A**) Bacterial concentrations: BC—bacterial control; BP MOI 1—bacteria plus phage at an MOI of 1; BP MOI 10—bacteria plus phage at an MOI of 10. (**B**) Phage concentration: PC MOI 1—phage control at an MOI of 1; PC MOI 10: phage control at an MOI of 10; PB MOI 1—phage and bacteria at an MOI of 1; PB MOI 10—phage and bacteria at an MOI of 10. The experiments were performed in TSB at 37 °C, and values represent the mean of three independent experiments. Error bars represent the standard deviation.

Bacterial kill curves were found using two different MOIs in TSB at 37 °C to determine whether increasing the phage dose would promote higher bacterial inactivation. In both assays, the bacterial control increased from 4.7 ± 0.16 × 10^5^ CFU/mL to 8.9 ± 0.14 × 10^8^ CFU/mL (ANOVA, *p* < 0.05, Figure 5A). When the bacterium was challenged with the phage at MOIs of 1 and 10, a maximum bacterial decrease of 4.9 and 5.4 log CFU/mL was observed after 9 h of incubation, respectively.

When higher doses of phage were used, it was observed that bacterial densities started to decrease earlier (after 6 h) compared to when an MOI of 1 was used (ANOVA, *p* < 0.05, Figure 5A). However, the maximum inactivation, observed after 9 h, did not present statistically significant differences (ANOVA, *p* > 0.05, Figure 5A). Nonetheless, when an MOI of 10 was used, the bacterial numbers remained constant for a more prolonged period (12–24 h), with statistically significant differences when compared with the MOI of 1 (ANOVA, *p* < 0.05, Figure 5A). Therefore, an MOI of 10 was selected for further studies.

Phage KP1LMA remained stable during this assay, with no variation in phage titer during the 24 h in the control group of both MOIs studied (Figure 5B). When phages were incubated in the presence of the host bacteria, a significant increase in phage titer of 2.9 and 1.7 log PFU/mL was observed for both test groups (ANOVA, *p* < 0.05, Figure 5B).

### 2.7. Bacterial Kill Curve in Urine

**Figure 6:**
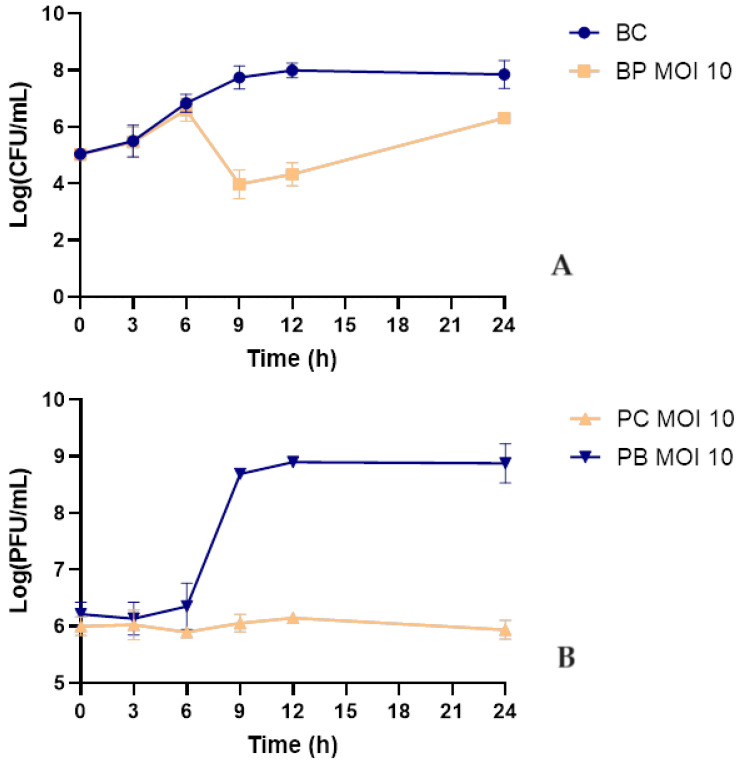
Inactivation of *K. pneumoniae* in sterile urine by phage KP1LMA using an MOI of 10. (**A**): bacterial concentrations; bacteria control—bacteria in the absence of phage; BP MOI 10—bacteria plus phage at an MOI of 10. (**B**): Phage concentration; PC MOI 10—phage control; PB MOI 10—phage in the presence of bacteria at a final MOI of 10. Values represent the mean of three independent experiments. Error bars represent the standard deviation.

Fresh, early morning urine was filtered using a 0.22 µm syringe filter and inoculated with phage KP1LMA and phage to a final MOI of 10. During the 24 h bacterial challenge, a maximum bacterial inactivation of 3.8 log CFU/mL was observed after 9 h (ANOVA, *p* < 0.05, Figure 6A). Despite bacterial regrowth, the differences in bacterial density between the test and control groups remained statistically significant until the end of the experiment (ANOVA, *p* < 0.05, Figure 6A). The phage remained stable with no significant differences in the control group (ANOVA, *p* > 0.05, Figure 6B). In the presence of the host, the phage titer increased significantly by 2.7 log PFU/mL (ANOVA, *p* < 0.05, Figure 6B).

### 2.8. Frequency of Emergence of Phage-Resistant Mutants 

When *K. pneumoniae* Scc 24 was challenged with phage KP1LMA, a frequency of emergence of phage-resistant mutants of 5.55 × 10^−3^ ± 0.002 was observed. After incubation for 24 h at 37 °C, a bacterial density of 4.51 × 10^8^ ± 0.61 CFU/mL was observed for the control group. However, only 2.52 × 10^4^ ± 0.93 CFU/mL was observed after 24 h when the bacteria were treated with phage.

## 3. Discussion

The rise of carbapenem-resistant *K. pneumoniae* poses a global public health threat [21,22]. Phages are increasingly considered an alternative or adjuvant treatment option, especially when antibiotics prove ineffective in treating bacterial infections [23,24,25,26]. Phage therapy is explored when patients do not respond to conventional antibiotic treatments [17]. However, it is still considered an experimental approach [27] and requires emergency regulatory approval [27], meaning that more data and research are needed to advance its integration into modern healthcare strategies. In the present study, phage KP1LMA was isolated using a strain of carbapenemase-producing *K. pneumoniae*, and its biological properties and in vitro efficacy were investigated. The results indicate that phage KP1LMA has the potential to control urinary tract infections caused by this strain of *K. pneumoniae*. Phage KP1LMA is an effective and safe phage capable of controlling a carbapenemase-producing *K. pneumoniae* strain in culture medium and urine that is not subject to inactivation at low pH. However, its narrow host range suggests that this phage will not be able to infect another strain. In most cases, it is necessary to use the pathogenic bacterium to isolate an effective phage for treatment or to test previously isolated phages in the libraries of different laboratories, which is the current procedure for phage alerts from the phage directory (https://phage.directory/alerts, accessed on 30 November 2023). Phage KP1LMA and other phages already isolated in different laboratories can be used worldwide in response to these alerts to inactivate *K. pneumoniae* strains involved in UTIs. However, due to this phage’s specificity, it would be more suitable to use in experimental procedures with the aim of generating novel broad-host-range phages. Nonetheless, this work results from an effort to expand our phage library to respond to phage alerts and to pave the way for more in-depth studies on the potential of bacteriophages to treat UTIs.

The genome of the *K. pneumoniae* phage KP1LMA is within the range of other described phages (size range 19,260–346,602 bp) [28], is a lytic phage and was classified as *Slopekvirus* (with a 176,096 bp linear double-stranded DNA). It does not appear to encode any known endolysin, toxin, or virulence or antibiotic resistance genes and can be considered safe for use in phage therapy. However, since this phage’s host was not sequenced, the presence of prophages cannot be excluded from the samples; therefore, the phage’s suspensions must be thoroughly screened for possible prophages prior to guaranteeing its safety for clinical application.

Although host specificity is considered one of the most advantageous properties of phages, distinguishing them from antibiotics, a narrow host range can be an obstacle to efficient phage therapy [9]. Phage KP1LMA showed a narrow host spectrum but can also infect *E. coli* ATCC 13706 as well as its host. Furthermore, clear lysis plaques formed in *E. coli* could still infect the *K. pneumoniae* host strain. Interestingly, phage plaques formed in *E. coli* were larger and generally clearer than those formed in the host (Figure S1). These observations could suggest that phage KP1LMA is closely related to the *E. coli* T4 phage. However, the efficiency of plating was considerably lower for *E. coli* than for the host strain *K. pneumoniae,* and genetic analysis showed a close relation to other *K. pneumoniae* phages. Therefore, other factors may affect lysis plaque formation [29,30], e.g., a long latent period. The same plaque morphology was observed for phage phi_KPN_S3, a phage from the same genus [31].

Our results suggest that the KP1LMA phage has the potential to control *K. pneumoniae* and *E. coli* ATCC 13706 strains, two closely related species of the *Enterobacteriaceae* family and the most predominant in UTIs [13]. The efficacy of phage KP1LMA was also tested in this study against other strains of *K. pneumoniae* and other bacterial genera of the *Enterobacteriaceae* family. However, the phage infected none of these bacteria. Generally, phages are highly specific, often infecting only one bacterial genus or even specific strains [9,10]. Shah et al. (2023) showed that phage RAM-1 infected only 3 of 16 *K. pneumoniae* strains tested [32]. Townsend and his coworkers (2021) isolated several new phages and observed that the MeTiny phage (*Slopekvirus*) presented the broadest host range, infecting 79% of all *Klebsiella* strains tested. However, the remaining phages identified as *Slopekvirus* presented a very narrow host range (forming plaques on one to two strains) [33]. Phage vB_kpnM_17-11 also showed a narrow host range, able to lyse only 4 out of 96 strains of *K. pneumoniae* tested [34]. In another study, phages LASTA and SJM3 infected only 5 *K. pneumoniae* of the 140 tested [24]. The narrow host range of phage KP1LMA may be due to bacterial resistance to phage adsorption, exopolysaccharide production, and/or capsule formation, which have been described for various bacteria [35]. In the future, efforts to broaden the phage’s host range may guarantee the clinical applicability of this therapy [36]. The most successful approach to improving a phage’s host range is to use the Appelman protocol [36]. However, this protocol requires an accessible phage library with multiple characterized phages in order to combine phage cocktails that will be used to generate novel phages with broad host range through recombination of prophages [36]. Therefore, in the future, phage KP1LMA can be used in combination with other phages to generate novel phages with a broad host range. Several studies have successfully produced new phages with broad host range using a combination of phages with a narrow host range, some only infecting two different strains [36,37,38,39,40]. Nonetheless, these phages should be studied against the clinical isolate before every application to provide a more specific treatment.

The success of phage therapy is generally attributed to parameters like adsorption rate and burst size [41]. The adsorption profile of phage KP1LMA showed that after 10 and 60 min, approximately 60 and 75% of the phage particles were adsorbed to the host cells, respectively, with an adsorption constant of K= 4.23246 × 10^−10^ mL^−1^ min^−1^. Similar results have been obtained with phage K2a [42]. However, in general, the adsorption rate of phage KP1LMA is lower than in other studies for *Klebsiella* phages [24,42,43]. Phage LASTA and SJM3 adsorption assays showed that approximately 97 and 94%, respectively, of the phage particles were adsorbed to *K. pneumoniae* after 20 min [24]. The adsorption constant is also considerably lower than what was observed by Balcão and his coworkers [44]. In a different study, Asghar and colleagues (2022) determined the adsorption constant for phages A¥L and A¥M (*Slopekvirus*) to be 4.4 × 10^−9^ and 5.1 × 10^−9^ mL/min, respectively [45]. A lower adsorption constant will result in fewer infected bacteria and a large population of uninfected bacteria remaining in contact with these free phages. This slow infection may allow for bacteria quorum sensing and increase the likelihood of triggering a phage defense mechanism, hindering therapeutic results [46]. Adjusting the phage concentrations to ensure maximum infection may improve the results [47].

Phage KP1LMA has a long latent period (100 min) and a small burst size (7.9 ± 0.3 PFU/host cell). It has been observed that *K. pneumoniae* phages have a wide range of latent periods and burst sizes, ranging from high burst sizes (410 PFU/infected cell) and short latent periods (21 min) [32] to low burst sizes (31.7 PFU/infected cell) and long latent periods (30 min) [34]. Baqer et al. (2022) demonstrated that *K. pneumoniae*-infecting phages K2a, K2b, K2w5, K2w6, Kp99, K9w5, K9w6, K9coc had burst sizes of 116, 41, 354, 106, 214, 66, 130 and 210 PFU/host cell, respectively, with latency periods of approximately 5, 20, 20, 25, 20, 30, 10 and 10 min, respectively [42]. Phage BL02, presented a burst size of 156 PFU/infected cell in an average lysis period of 50 min [48]. Phages LASTA and SJM3 presented a higher burst size of 187 ± 37 and 155 ± 34 PFU/host cell, respectively, and a long latent period of 80 min [24]. In another study, phage HS106 presented higher burst size (183 PFU/host cell) and low latent period (10 min) [43]. However, the phage’s burst size is also affected by the host, as observed by Kondo (2023) [49]. Although phage KP1LMA presents a small burst size (7.9 ± 0.3 PFU/host cell) and a long latency period (100 min) (Figure 4), phages replicate efficiently in the host, causing a high reduction in *K. pneumoniae* growth, suggesting that other factors regulate the phage–bacteria interaction. Nonetheless, a prolonged latent period may negatively affect the phage’s inactivation potential since secondary adsorptions may occur in a phenomenon described as lysis inhibition [50]. However, since this phage presented a low adsorption rate, the likelihood of secondary infections is unlikely, and this may be a characteristic that favors application in scenarios of bacterial biofilm formation, as is the case for UTIs [51]. A phage’s burst size and latent period depend on the phage type, the host’s physiological state, the growth medium’s composition, the pH, and the temperature of the incubation [42]. A smaller burst size can be caused by the phage’s large size and the host’s small size. The host cell’s size is critical as it modulates the availability of receptors and its protein synthesis machinery for phage binding and growth [52]. Larger burst sizes and longer latent periods increase the likelihood of successful dispersal in the environment [53], indicating that the phage is widely available for isolation. For phage KP1LMA, the long latent period did not result in a large burst size, which may indicate that the eclipse time of this phage is considerably longer than normal. In the future, it will be important to characterize this infection parameter to better understand this infection. According to Abedon and coworkers [54], these results may suggest that the isolated phage is not specific to the tested strain. However, of the two bacterial strains this phage could infect, the phage presented a higher affinity towards *Klebsiella* than *E. coli*. Since the genetic analysis showed similarity to other *Klebsiella* phages, perhaps other *Klebsiella* stains will prove more susceptible to this phage and provide better overall results.

*Klebsiella pneumoniae* was effectively inactivated by KP1LMA phage in TSB medium (with a maximum inactivation of 5.4 log CFU/mL after 9 h). The increase in the MOI from 1 to 10 increased treatment efficiency. The maximum inactivation of phage KP1LMA at MOI 1 (with a maximum inactivation of 4.9 log CFU/mL) and 10 (5.4 log CFU/mL) was statistically similar (Figure 5A). However, when an MOI of 10 was used, bacterial regrowth was delayed, and the bacterial concentration remained constant until the end of the experiment. Chen et al. (2023) also observed that lower phage concentrations can induce bacterial regrowth more rapidly than those treated with higher concentrations [43]. The authors observed that bacterial density (OD600) increased more rapidly when incubated at a lower MOI of 1 compared to higher MOIs (10 and 100) [43]. Notably, despite the phage showing a life cycle of 100 min (between adsorption and burst), the decrease in bacterial concentrations was only observed after about 3 to 6 h in the inactivation assay. This result was also observed for phage VB_EcoM-Sa45lw, which presented a latent period of 30 min, yet bacterial decrease was only observed after 4 to 6 h [55]. A similar result was observed by Lam and coworkers (2023) for phages TCUAN1 and TCUAN2, which, despite a life cycle of about 80 and 30 min and adsorption of almost 100% in 4 and 2 min, bacterial inactivation was only observed after 60 to 240 min, respectively [56]. The number of phage particles of KP1LMA when incubated with *K. pneumoniae* for 12 h at an MOI of 1 and 10 increased by 2.7 and 1.9 log PFU/mL, respectively. These results demonstrate that high initial phage doses may not be essential due to the self-perpetuating nature revealed by increasing phage titer and bacteria.

The efficiency of phage KP1LMA was tested in human urine samples to evaluate the potential application of this phage for the inactivation of UTI caused by *K. pneumoniae*. It was observed that phage KP1LMA could successfully inactivate *K. pneumoniae* in urine (maximum inactivation of 3.8 log CFU/mL). The concentration of phage KP1LMA remained constant in the absence of the host. Its titer increased significantly in the presence of the bacterium during the experiment. However, its effectiveness in inactivating *K. pneumoniae* in urine (maximum concentration of 3.8 log CFU/mL after 9 h of incubation) was significantly lower compared to experiments in TSB medium (maximum inactivation of 5.4 log CFU/mL after 9 h of incubation). Although the phage concentration remained constant throughout the experiment and phage KP1LMA replication in the presence of *K. pneumoniae* was similar in TSB medium and urine (2.9 and 2.7 log PFU/mL, respectively), the lower bacterial inactivation in urine could be due to lower phage viability. Silva et al. (2014) showed that although phages survive at different pH values, their efficiency in inactivating bacteria is affected by low pH values [57]. In general, phages’ lytic activity decreases at pH values 10 < pH < 5, with optimum pH conditions around neutrality (pH of 6–8) [58,59]. Similar results were obtained by Pereira et al. (2016) [60]. These authors showed that single-phage suspensions of phages E-2 and E-4 and the phage cocktail E-2/E-4 reduced approximately 2.0 log CFU/mL of *Enterobacter cloacae* in urine. The efficacy of the single-phage suspensions and phage cocktail was lower than that of phosphate-buffered saline (PBS), which reduced 3.4 log CFU/mL [60]. Further studies using the phage KP1LMA as a model in urine should be carried out to understand the impact of urine on bacterial metabolic functions and phage infectivity [61].

A major concern regarding bacterial inactivation by phages is the emergence of phage-resistant bacteria [62,63,64,65]. The development of resistant mutants, which only occurred in bacteria exposed to the KP1LMA phage, was limited (5.55 × 10^−3^). These results are in close agreement with results obtained by other researchers [65,66,67,68]. Phage cocktails can help overcome the problem of bacterial phage resistance during therapeutical applications [59]. However, their success requires that phages use different infection mechanisms and are not affected by cross-resistance [69]; this may be achieved by using phages that use different bacterial receptors. 

The results of this study show that the newly isolated phage can effectively inactivate a strain of *K. pneumoniae* in urine. Furthermore, the phage is also safe to use, as it is strictly lytic without known genes encoding for virulence or antibiotic resistance. However, as the viral infection is very specific, continuing this phage’s isolation and characterization effort is important for the delivery of safe alternatives. Nonetheless, despite its inability to infect other *Klebsiella* strains, this phage is a suitable candidate for evolutionary experiments with the aim of broadening host range. Therefore, efforts must be made to continue broadening phage libraries in order to find suitable and safe candidates that can answer any request for phages for compassionate treatment. 

## 4. Materials and Methods

### 4.1. Bacterial Strains and Growth Conditions

The bacterial strains used in this study are listed in Table 1. The bacterial strain *K. pneumoniae* Scc 24 was previously isolated [70] and used as a phage host. *Escherichia coli* (ATCC 13706 and ATCC 25922), *S.* Typhimurium (ATCC 13311 and ATCC 14028), and *S. flexneri* DSM 4782 were purchased from the ATCC and DSM collections, respectively. *Enterobacter cloacae* was previously isolated by our group [60]. Five strains of *S.* Enteritidis were isolated from food and provided by Controlvet Laboratory. The other bacterial strains used in this study were isolated in other studies [60,70,71]. Fresh bacterial cultures were kept at 4 °C in Tryptic Soy Agar (TSA, Liofilchem, Roseto degli Abruzzi, Italy). Before each assay, one isolated colony was transferred to 30 mL of TSB and grown overnight at 37 °C with orbital shaking (120 rpm) until an optical density (O.D. 600) of 0.8, corresponding to about 10^9^ cells/mL.

### 4.2. Phage Isolation and Purification

Phage KP1LMA was isolated from the sewage network of Aveiro (station EEIS9 of SIMRIA Multi Sanitation System of Ria de Aveiro). About 50 milliliters of water was filtered through polycarbonate membranes with a 0.45 µm pore size (Millipore, Bedford, MA, USA) and added 50 mL of a twice-concentrated TSB medium. The mixture was inoculated with 1 mL of *K. pneumoniae* Scc 24 in the exponential phase and incubated for 24 h at 25 °C and 120 rpm. After incubating, the solution was centrifuged at 10,000× *g* for 10 min at 4 °C and filtered through a polyethersulfone layer with a 0.22 µm pore size (Merck-Millipore, Darmstadt, Germany). The filtrate was stored at 4 °C, and the titer was determined according to [72]. Serial dilutions of the filtrate stock were performed in phosphate-buffered saline (PBS) [137 mmol^−1^ NaCl (Sigma, St. Louis, MO, USA), 8.1 mmol^−1^ Na_2_HPO_4_·2H_2_O (Sigma, St. Louis, MO, USA), 2.7 mmol^−1^ KCl (Sigma, St. Louis, MO, USA), and 1.76 mmol^−1^ KH_2_PO_4_ (Sigma, St. Louis, MO, USA), pH 7.4). Then, 500 microlitres of each dilution, along with 200 µL of fresh bacterial culture, were added to 5 mL of molten TSB 0.6% top agar layer [30 g/L TSB (Liofilchem, Roseto degli Abruzzi, Italy), 6 g/L agar (Liofilchem, Roseto degli Abruzzi, Italy), 0.12 g/L MgSO_4_ (Sigma, St. Louis, MO, USA), and 0.05 g/L CaCl_2_ (Sigma, St. Louis, MO, USA), pH 7.4] and poured over a TSA plate. Plates were incubated at 37 °C and observed for the presence of lytic plaques after 16 to 18 h. One single plaque was selected from the solid medium and added to the TSB medium with a fresh culture of the host. The sample was centrifuged, and the supernatant was used as a phage source for a second isolation procedure. Three successive single-plaque isolation cycles were performed to acquire pure phage stock. All lysates were centrifuged at 10,000× *g* for 10 min at 4 °C to remove bacteria or bacterial debris. The phage suspensions were kept at 4 °C.

### 4.3. Electron Microscope Examination

Phage particles of a highly concentrated suspension (10^9^ PFU/mL) were negatively stained with uranyl acetate (Electron Microscopy Sciences, Hatfield, PA, USA). Briefly, 15 µL of each sample was adsorbed to carbon-coated collodion 400-mesh nickel grids (Fisher Scientific, Hampton, VA, USA) for 2 min and stained with 2% aqueous uranyl acetate (Electron Microscopy Sciences) for 1 min. Grids were visualized in a JEOL JEM-1011 transmission electron microscope (operating at 100 kV). Micrographs were taken with a MEGA VIEW III (SIS) digital camera at various magnifications.

### 4.4. Phage DNA Extraction

The extraction of the virion’s nucleic acid was performed according to Jakočiūnė and Moodley (2018) [73], with bacterial DNA and RNA removal and digestion of the bacteriophage capsid [74] and DNA purification performed with a DNeasy Blood & Tissue Kit (Qiagen, Hilden, Germany). The quantity and quality of the DNA were measured in a NanoDrop One UV–Vis scanning spectral microdroplet spectrophotometer (ThermoScientific, Waltham, MA, USA), according to the manufacturer’s instructions.

### 4.5. Phage Genome Assembly and Annotation

Phage genome sequencing was performed by SeqCenter, LLC (Pittsburgh, PA 15201, Est (Lisboa, Portugal) using an Illumina MiSeq with 300 bp paired-end reads. The library was constructed using a Kit Kapa HyperPlus according to the manufacturer’s protocol. 

The quality control of raw sequence reads was performed using FASTQC v0.11.9 (FastQC source: Bioinformatics Group at the Babraham Institute, UK) before and after trimming low-quality reads with Trimmomatic v.0.39 with the following parameters: ILLUMINACLIP–adaptors–fasta: 2:30:1, Leading: 8, Trailing: 8, Slidingwindow: 4:15, and Minlen: 100 [75]. The trimmed reads were subjected to de novo genome assembly using SPAdes v.3.13.1 with careful parameters [76]. The assembly graph was inspected using Bandage v0.8.1 [77]. The reads were then mapped back against the resulting assembly using BBMap v.38.18 to determine the average coverage of each contig [78]. Manual filtering was performed to remove contigs with dubious coverage. The reads were mapped back to the filtering contig file using Bowtie2 v.2.5.1 [79] and assembled with SPAdes v.3.13.1. Further assembly error correction and polishing were performed using Pilon v1.24 [80]. Termini could not be predicted using PhageTerm [81]; however, the genome was found to be circularly permuted using apc.pl (https://github.com/jfass/apc, accessed on 6 October 2023), and the repeated sequence artifacts were removed. The phage genome was manually reordered to match the most closely related phage based on the method reported by Shen and Millard [82]. The full-genome sequence of the KP1LMA phage was compared to the phage genome sequences in GenBank using BLASTN (somewhat similar sequences) in the NCBI database, and the most closely related phages were identified. The completeness and contamination of the phage genome sequence were tested using CheckV v1.0.1 [83]. The phage genome was annotated for coding DNA sequences (CDS), tRNA, tmRNA, CRISPRs, virulence factors (VFs), toxins, and antimicrobial resistance genes (ARGs) using Pharokka v1.3.2 [84], and the CDS were assigned to functional categories using PHROGs [85].

### 4.6. Phage Host Range and Efficiency of Plating 

The phage host range was assessed through spot-testing according to [72] for the bacterial strains in Table 1. Briefly, molten-top 0.6% agar was inoculated with 300 µL of fresh exponential bacteria culture, poured over a TSA plate, and allowed to dry. Subsequently, 50 to 100 µL of phage stock was spotted over the bacterial lawn and plates were incubated at 37 °C for 16 to 18 h before observation. A clear lysis zone on the area where the phage had been spotted would indicate sensitivity to the phage. Bacterial sensitivity to phage infection was recorded according to a clear lysis zone (x) or absence of lysis zone (-) (Table 1). Additionally, phage plaques formed on different bacterial strains were used for isolation and cross-infection against the original *K. pneumoniae* host bacterium in order to prove that the infection is caused by the *K. pneumoniae* phage and not by any temperate phages eventually presented in the genome of these other bacteria.

### 4.7. Adsorption Curve 

The phage adsorption curve was determined according to [47,72]. Briefly, phages (10^3^ PFU/mL) were added to a mid-exponential bacterial culture (cell density of about 10^6^ CFU/mL) at 37 °C in TSB. Every 10 min, an aliquot was collected, a few drops of chloroform were added, and the sample was centrifuged at 10,000× *g* for 5 min at 4 °C. The number of unadsorbed phage particles was determined by plating the sample using the double-agar-layer technique [72]. The percentage of adsorbed phages was calculated by comparing the free phage titer at a given point with the initial phage titer. Three independent assays were performed. The adsorption constant was calculated according to [86] using the following formula:

$$k=\left(-\frac{1}{Bt}\right)\times \ln\left(\frac{P}{P0}\right)$$

*P*—concentration of free phage per mL, *P*0—initial concentration of phage, *B*—initial concentration of bacteria, *k*—adsorption rate constant (mL/min), *t*—time (min).

### 4.8. One-Step Growth Assay

Phage suspension (final concentration of 10^6^ PFU/mL) was added to 10 mL of fresh bacterial culture of *K. pneumoniae* (with a final concentration of 10^9^ PFU/mL) to obtain a final MOI of 0.001 and incubated for 5 min at 37 °C without shaking. The suspension was then centrifuged at 10,000× *g* for 5 min, the supernatant was discarded, and the pellet was resuspended in 10 mL of TSB and incubated at 37 °C according to the method of Hyman and Abedon [47]. A 1 mL sample was collected and immediately titered using the double-agar-layer technique. Plates were incubated at 25 °C and observed after 18 h. Samples were collected every 20 min for 140 min, and three independent assays were performed.

### 4.9. Bacterial Kill Curve in TSB Culture Media

To characterize the bacterial kill curves, 30 mL of sterile TSB was inoculated with the strain *K. pneumoniae* Scc 24, which was then challenged with phage KP1LMA at MOIs of 1 and 10 (final concentration 10^5^ and 10^6^ PFU/mL, respectively). Two controls were included in this assay: the bacterial control (BC) and the phage control (PC). The bacterial control was inoculated with *K. pneumoniae* but not with phage, and the phage control was inoculated only with phage. Controls and test samples were kept in the same conditions (37 °C and no agitation). Aliquots were collected from all controls and samples at 0, 3, 6, 9, 12, and 24 h of incubation. Phage titer was determined in duplicate by the double agar layer [72] after an incubation period of 16–18 h at 37 °C. Bacterial concentration was determined in duplicate in the TSA medium after an incubation period of 24 h at 37 °C. Three independent assays were performed for each MOI.

### 4.10. Bacterial Kill Curve in Urine Samples

#### 4.10.1. Urine Sample Collection and Handling

According to the method of Pereira and coworkers [60], early urine samples were collected using a post-hygiene, midstream clean-catch technique. After performing daily hygiene, the micturition middle stream was collected directly into sterile containers after discarding the initial portion. The end portions were also discarded. The samples were immediately transported to the laboratory, centrifuged at 10,000× *g* for 10 min and filtered through a 0.22 µm pore size (Merck-Millipore, Darmstadt, Germany) into sterile containers to remove bacteria eventually present in the urine samples. The urine samples were always collected from the same person at the same hours.

#### 4.10.2. Bacterial Kill Curves in Urine

The efficacy of the phage KP1LMA in controlling *K. pneumoniae* was assessed in the sterile urine for an MOI of 10 to evaluate the possible application of this phage during a UTI. An Erlenmeyer flask containing 30 mL of sterile urine was inoculated with bacteria (final concentration of 10^5^ CFU/mL) and phage (final concentration of 10^6^ PFU/mL). Two other flasks were included as control groups. The phage control group was only inoculated with phage (PC), and the bacteria control group was only inoculated with bacteria without the phage (BC). The flasks were incubated at 37 °C with no agitation, and aliquots were collected after 0, 3, 6, 9, 12 and 24 h. Bacterial concentration was determined, in duplicate, in the TSA plate after an incubation period of 24 h. In duplicate, the double-agar-layer method [72] determined phage titer after an incubation period of 24 h at 37 °C. Three independent assays were performed.

### 4.11. Rate of Emergence of Phage-Resistant Bacterial Mutants

The emergence rate of phage-resistant bacterial mutants was determined according to the method of Filippov et al., 2011. Ten phage-sensitive colonies were isolated from a TSA plate and incubated at 37 °C in test tubes containing 5 mL of TSB medium for 24 h (final concentration of about 10^9^ CFU/mL). Each bacterial culture was serially diluted, and aliquots of 100 µL from the 10^0^ to 10^−2^ dilutions were transferred to molten-top TSB 0.6% agar previously inoculated with 100 µL of phage (from a phage stock with a final titer of about 10^9^ PFU/mL) and poured over a TSA plate. The plates were incubated at 37 °C for 3–5 days as some mutants grow slowly. In parallel, an aliquot of 100 µL of the 10^−5^ to 10^−7^ dilutions was incorporated on TSA plates without the addition of phage and incubated for 24 h at 37 °C. The frequency of spontaneous phage-resistant mutation was calculated by dividing the number of resistant bacteria (colonies counted in the presence of phage) by the total number of sensitive bacteria (CFU counted in the plates without phage) and multiplied by 100. For this assay, three independent experiments were performed.

### 4.12. Statistical Analysis

Statistical analysis was performed using GraphPad Prism software 8.4.3 (San Diego, CA, USA). Normal distribution of the data was assessed by the Kolmogorov–Smirnov test and the homogeneity of variance was confirmed by Levene’s test. A two-way ANOVA with repeated measures and Tukey’s multiple comparisons post hoc test were used to study the significance of bacterial and viral concentrations between treatments and during the experiments. A *p*-value below 0.05 was considered statistically significant.

## 5. Conclusions

The results indicate that phage KP1LMA could potentially control a UTI caused by this strain of *K. pneumoniae*, suggesting that the same procedure can be used to control UTIs caused by other strains if new phages specific to those strains are isolated. Phage KP1LMA is an effective and safe phage that can control carbapenemase strain-producing *K. pneumoniae* in culture medium and urine and does not suffer inactivation at low pH. Although phage KP1LMA is highly specific, in the future, efforts can be made to expand its spectrum of activity and to combine this phage with others in cocktails, potentially enabling its use against other *K. pneumoniae* strains involved in UTIs.

## Figures and Tables

**Table 1:** Phage host range and efficiency of plating on 52 different bacterial strains.

Bacterial Strains	Phage KP1LMA	
	Spot Test	EOP (%)
*Klebsiella pneumoniae* Scc 24	+	100
*Klebsiella pneumoniae* Scc 1	−	0
*Klebsiella pneumoniae* Scc 5	−	0
*Klebsiella pneumoniae* Scc 11	−	0
*Klebsiella pneumoniae* Scc 15	−	0
*Klebsiella pneumoniae* Scc 17	−	0
*Escherichia coli* ATCC 13706	+	32.98 ± 2.85
*Escherichia coli* ATCC 25922	−	0
*Escherichia coli* Scc 9	−	0
*Escherichia coli* Scc 33	−	0
*Escherichia coli* Scc 34	−	0
*Escherichia coli* Scc 35	−	0
*Escherichia coli* Scc 36	−	0
*Escherichia coli* Scc 37	−	0
*Escherichia coli* Scc 39	−	0
*Escherichia coli* Scc 40	−	0
*Escherichia coli* Scc 41	−	0
*Escherichia coli* Scc 43	−	0
*Escherichia coli* Scc 45	−	0
*Escherichia coli* Scc 47	−	0
*Escherichia coli* Scc 48	−	0
*Escherichia coli* Scc 49	−	0
*Escherichia coli* Scc 51	−	0
*Escherichia coli* Scc 52	−	0
*Escherichia coli* Scc 53	−	0
*Escherichia coli* Scc 55	−	0
*Escherichia coli* Scc 56	−	0
*Escherichia coli* Scc 58	−	0
*Escherichia coli* Scc 69	−	0
*Escherichia coli* Scc 77	−	0
*Escherichia coli* Scc 78	−	0
*Escherichia coli* Scc 91	−	0
*Escherichia coli* Scc 98	−	0
*Escherichia coli* BC30	−	0
*Escherichia coli* AE11	−	0
*Escherichia coli* AD6	−	0
*Escherichia coli* AF15	−	0
*Escherichia coli* AN19	−	0
*Escherichia coli* AC5	−	0
*Escherichia coli* AJ23	−	0
*Escherichia coli* BN65	−	0
*Escherichia coli* BM62	−	0
*Citrobacter freundii* 6F	−	0
*Enterobacter cloacae*	−	0
*Providencia* sp.	−	0
*Salmonella enterica serovar Enteriditis CVA*	−	0
*Salmonella enterica serovar Enteriditis CVB*	−	0
*Salmonella enterica serovar Enteriditis CVC*	−	0
*Salmonella enterica serovar Enteriditis CVD*	−	0
*Salmonella enterica serovar Enteriditis CVE*	−	0
*Salmonella enterica serovar* Typhimurium ATCC 14028	−	0
*Salmonella enterica serovar* Typhimurium ATCC 13311	−	0
*Shigella flexneri* DSM 4782	−	0

## Data Availability

Data are contained within the article and supplementary materials.

## Supplementary Materials

The following supporting information can be downloaded at: https://www.mdpi.com/article/10.3390/antibiotics13020195/s1. Table S1. Coding sequences identified in KP1LMA phage. Table S2. Number of CDSs assigned to functional categories using pharokka. Figure S1. Plaques formed on *E. coli* ATCC 13706 (A) were completely clear, whereas plaques formed on the original host (B) presented a turbid characteristic and were smaller.
