# Supplementary material for: Potential of an Isolated Bacteriophage to Inactivate Klebsiella pneumoniae: Preliminary Studies to Control Urinary Tract Infections
Source: Antibiotics (Basel). 2024 Feb 19;13(2):195. doi: 10.3390/antibiotics13020195 (PMC10885952; doi:10.3390/antibiotics13020195)
Supplement: Supplementary file 1 [file antibiotics-13-00195-s001.zip › antibiotics-2827544-supplementary.pdf]

Table S1. Coding sequences identified in KP-1 phage.

gene	start	stop	frame	Region	Putative function	PHROGs category
OJRRIMKN_0001	158	3	-	CDS	starvation-inducible transcriptional regulator	transcription regulation
OJRRIMKN_0002	411	130	-	CDS	lysis inhibition	lysis
OJRRIMKN_0003	910	518	-	CDS	hypothetical protein	unknown function
OJRRIMKN_0004	1535	927	-	CDS	thymidine kinase	other
OJRRIMKN_0005	1725	1528	-	CDS	hypothetical protein	unknown function
OJRRIMKN_0006	2005	1718	-	CDS	hypothetical protein	unknown function
OJRRIMKN_0007	2481	2008	-	CDS	phosphatase	other
OJRRIMKN_0008	2659	2471	-	CDS	hypothetical protein	unknown function
OJRRIMKN_0009	2930	2814	-	CDS	hypothetical protein	unknown function
OJRRIMKN_0010	3253	2930	-	CDS	valyl tRNA synthetase modifier	DNA, RNA and nucleotide metabolism
OJRRIMKN_0011	3853	3263	-	CDS	hypothetical protein	unknown function
OJRRIMKN_0012	3996	3850	-	CDS	hypothetical protein	unknown function
OJRRIMKN_0013	4402	4007	-	CDS	hypothetical protein	unknown function
OJRRIMKN_0014	5111	4470	-	CDS	unknown function	unknown function
OJRRIMKN_0015	5730	5176	-	CDS	hypothetical protein	unknown function
OJRRIMKN_0016	5914	5732	-	CDS	hypothetical protein	unknown function
OJRRIMKN_0017	6120	5923	-	CDS	hypothetical protein	unknown function
OJRRIMKN_0018	7504	6158	-	CDS	PhoH-like phosphate starvation-inducible	other
OJRRIMKN_0019	8038	7514	-	CDS	HNH endonuclease	DNA, RNA and nucleotide metabolism
OJRRIMKN_0020	8496	8038	-	CDS	hypothetical protein	unknown function
OJRRIMKN_0021	8923	8528	-	CDS	endolysin	lysis
OJRRIMKN_0022	9392	8991	-	CDS	hypothetical protein	unknown function
OJRRIMKN_0023	9770	9447	-	CDS	hypothetical protein	unknown function
OJRRIMKN_0024	10073	9810	-	CDS	hypothetical protein	unknown function
OJRRIMKN_0025	10403	10143	-	CDS	hypothetical protein	unknown function
OJRRIMKN_0026	10836	10405	-	CDS	hypothetical protein	unknown function
OJRRIMKN_0027	11100	10846	-	CDS	hypothetical protein	unknown function
OJRRIMKN_0028	11287	11114	-	CDS	hypothetical protein	unknown function
OJRRIMKN_0029	11631	11284	-	CDS	hypothetical protein	unknown function
OJRRIMKN_0030	11956	11612	-	CDS	hypothetical protein	unknown function
OJRRIMKN_0031	13107	11953	-	CDS	hypothetical protein	unknown function
OJRRIMKN_0032	13120	13848	+	CDS	homing endonuclease	DNA, RNA and nucleotide metabolism
OJRRIMKN_0033	14095	13841	-	CDS	hypothetical protein	unknown function

OJRRIMKN_0034	14241	14095	-	CDS	hypothetical protein	unknown function
OJRRIMKN_0035	14489	14238	-	CDS	hypothetical protein	unknown function
OJRRIMKN_0036	14839	14561	-	CDS	hypothetical protein	unknown function
OJRRIMKN_0037	15180	14923	-	CDS	hypothetical protein	unknown function
OJRRIMKN_0038	15755	15180	-	CDS	hypothetical protein	unknown function
OJRRIMKN_0039	16161	15763	-	CDS	hypothetical protein	unknown function
OJRRIMKN_0040	16536	16336	-	CDS	hypothetical protein	unknown function
OJRRIMKN_0041	17527	16613	-	CDS	hypothetical protein	unknown function
OJRRIMKN_0042	18051	17524	-	CDS	hypothetical protein	unknown function
OJRRIMKN_0043	18374	18051	-	CDS	hypothetical protein	unknown function
OJRRIMKN_0044	18660	18403	-	CDS	hypothetical protein	unknown function
OJRRIMKN_0045	19045	18662	-	CDS	hypothetical protein	unknown function
OJRRIMKN_0046	19463	19056	-	CDS	nudix hydrolase	other
OJRRIMKN_0047	19709	19464	-	CDS	hypothetical protein	unknown function
OJRRIMKN_0048	19957	19706	-	CDS	hypothetical protein	unknown function
OJRRIMKN_0049	20243	19950	-	CDS	hypothetical protein	unknown function
OJRRIMKN_0050	20575	20240	-	CDS	hypothetical protein	unknown function
OJRRIMKN_0051	20726	20586	-	CDS	hypothetical protein	unknown function
OJRRIMKN_0052	21561	21022	-	CDS	hypothetical protein	unknown function
OJRRIMKN_0053	22192	21569	-	CDS	RNA ligase	DNA, RNA and nucleotide metabolism
OJRRIMKN_0054	22516	22244	-	CDS	tail fiber chaperone	tail
OJRRIMKN_0055	23214	22519	-	CDS	deoxynucleoside monophosphate kinase	other
OJRRIMKN_0056	23744	23214	-	CDS	tail tube	tail
OJRRIMKN_0057	24114	24902	+	CDS	hypothetical protein	unknown function
OJRRIMKN_0058	25793	24939	-	CDS	DNA end protector	DNA, RNA and nucleotide metabolism
OJRRIMKN_0059	26276	25809	-	CDS	head closure	connector
OJRRIMKN_0060	26327	26881	+	CDS	baseplate wedge subunit	tail
OJRRIMKN_0061	26878	28647	+	CDS	baseplate hub subunit and tail lysozyme	tail
OJRRIMKN_0062	28649	30715	+	CDS	hypothetical protein	unknown function
OJRRIMKN_0063	30747	32672	+	CDS	baseplate wedge subunit	tail
OJRRIMKN_0064	32753	35839	+	CDS	baseplate wedge subunit	tail
OJRRIMKN_0065	35840	36832	+	CDS	baseplate wedge subunit	tail
OJRRIMKN_0066	36842	37705	+	CDS	baseplate wedge tail fiber protein connector	tail
OJRRIMKN_0067	37702	39519	+	CDS	baseplate wedge subunit	tail
OJRRIMKN_0068	39519	40187	+	CDS	baseplate wedge subunit	tail

OJRRIMKN_0069	40197	41582	+	CDS	tail collar fiber protein	tail
OJRRIMKN_0070	41596	43347	+	CDS	fibritin neck whisker	tail
OJRRIMKN_0071	43386	44312	+	CDS	head-tail adaptor Ad2	connector
OJRRIMKN_0072	44322	45068	+	CDS	head closure Hc2	connector
OJRRIMKN_0073	45149	45973	+	CDS	tail sheath stabilizer	tail
OJRRIMKN_0074	45973	46512	+	CDS	terminase small subunit	head and packaging
OJRRIMKN_0075	46481	48310	+	CDS	terminase large subunit	head and packaging
OJRRIMKN_0076	48330	50321	+	CDS	tail sheath	tail
OJRRIMKN_0077	50370	50855	+	CDS	tail tube	tail
OJRRIMKN_0078	50911	52485	+	CDS	portal protein	head and packaging
OJRRIMKN_0079	52485	52727	+	CDS	prohead	head and packaging
OJRRIMKN_0080	52736	53140	+	CDS	head scaffolding protein	head and packaging
OJRRIMKN_0081	53143	53790	+	CDS	head maturation protease	head and packaging
OJRRIMKN_0082	53823	54611	+	CDS	head scaffolding protein	head and packaging
OJRRIMKN_0083	54631	56199	+	CDS	major head protein	head and packaging
OJRRIMKN_0084	56285	56920	+	CDS	homing endonuclease	DNA, RNA and nucleotide metabolism
OJRRIMKN_0085	56953	58242	+	CDS	major head protein	head and packaging
OJRRIMKN_0086	58547	58966	+	CDS	UvsY-like recombination mediator	DNA, RNA and nucleotide metabolism
OJRRIMKN_0087	58966	59142	+	CDS	unknown function	unknown function
OJRRIMKN_0088	59810	59577	-	CDS	DNA helicase	DNA, RNA and nucleotide metabolism
OJRRIMKN_0089	61318	59819	-	CDS	DNA helicase	DNA, RNA and nucleotide metabolism
OJRRIMKN_0090	61389	62012	+	CDS	minor head protein inhibitor of protease	head and packaging
OJRRIMKN_0091	62105	62416	+	CDS	hypothetical protein	unknown function
OJRRIMKN_0092	62456	63025	+	CDS	hypothetical protein	unknown function
OJRRIMKN_0093	63054	64115	+	CDS	tail protein	tail
OJRRIMKN_0094	64126	64401	+	CDS	Hoc-like head decoration	head and packaging
OJRRIMKN_0095	64415	64933	+	CDS	5'-3' deoxyribonucleotidase	moron, auxiliary metabolic gene and host takeover
OJRRIMKN_0096	65429	64965	-	CDS	5'-3' deoxyribonucleotidase	moron, auxiliary metabolic gene and host takeover
OJRRIMKN_0097	66318	65479	-	CDS	hypothetical protein	unknown function
OJRRIMKN_0098	66433	67308	+	CDS	hypothetical protein	unknown function
OJRRIMKN_0099	67341	67658	+	CDS	hypothetical protein	unknown function
OJRRIMKN_0100	67655	67831	+	CDS	hypothetical protein	unknown function
OJRRIMKN_0101	67828	68154	+	CDS	hypothetical protein	unknown function
OJRRIMKN_0102	68156	68512	+	CDS	hypothetical protein	unknown function
OJRRIMKN_0103	68512	69036	+	CDS	decoy of host sigma70	moron, auxiliary metabolic gene and host takeover

OJRRIMKN_0104	69040	70059	+	CDS	RNA ligase	DNA, RNA and nucleotide metabolism
OJRRIMKN_0105	70049	70630	+	CDS	DprA-like DNA recombination-mediator protein	DNA, RNA and nucleotide metabolism
OJRRIMKN_0106	70699	71142	+	CDS	hypothetical protein	unknown function
OJRRIMKN_0107	71220	71480	+	CDS	hypothetical protein	unknown function
OJRRIMKN_0108	71490	71939	+	CDS	hypothetical protein	unknown function
OJRRIMKN_0109	71967	73400	+	CDS	nicotinamide phosphoribosyl transferase	other
OJRRIMKN_0110	73387	73689	+	CDS	hypothetical protein	unknown function
OJRRIMKN_0111	73686	74537	+	CDS	DNA methyltransferase	other
OJRRIMKN_0112	74546	74821	+	CDS	hypothetical protein	unknown function
OJRRIMKN_0113	74853	75335	+	CDS	endonuclease VII	DNA, RNA and nucleotide metabolism
OJRRIMKN_0114	75332	77455	+	CDS	NrdD-like anaerobic ribonucleotide reductase large subunit	moron, auxiliary metabolic gene and host takeover
OJRRIMKN_0115	77523	77765	+	CDS	hypothetical protein	unknown function
OJRRIMKN_0116	77762	78256	+	CDS	hypothetical protein	unknown function
OJRRIMKN_0117	78256	78462	+	CDS	hypothetical protein	unknown function
OJRRIMKN_0118	78483	78734	+	CDS	hypothetical protein	unknown function
OJRRIMKN_0119	78795	78986	+	CDS	hypothetical protein	unknown function
OJRRIMKN_0120	79013	79654	+	CDS	homing endonuclease	DNA, RNA and nucleotide metabolism
OJRRIMKN_0121	79651	80142	+	CDS	anaerobic ribonucleotide reductase small subunit	DNA, RNA and nucleotide metabolism
OJRRIMKN_0122	80212	81171	+	CDS	hypothetical protein	unknown function
OJRRIMKN_0123	81214	81882	+	CDS	hypothetical protein	unknown function
OJRRIMKN_0124	81842	82123	+	CDS	hypothetical protein	unknown function
OJRRIMKN_0125	82120	82419	+	CDS	hypothetical protein	unknown function
OJRRIMKN_0126	82499	86272	+	CDS	tail fiber protein proximal subunit	tail
OJRRIMKN_0127	86272	87396	+	CDS	long tail fiber protein proximal connector	tail
OJRRIMKN_0128	87446	88114	+	CDS	hinge connector of long tail fiber protein distal connector	tail
OJRRIMKN_0129	88123	92103	+	CDS	tail fiber protein	tail
OJRRIMKN_0130	92140	92667	+	CDS	tail fiber adhesin	tail
OJRRIMKN_0131	92784	93431	+	CDS	holin	lysis
OJRRIMKN_0132	93701	93432	-	CDS	hypothetical protein	unknown function
OJRRIMKN_0133	93988	93698	-	CDS	hypothetical protein	unknown function
OJRRIMKN_0134	94785	93988	-	CDS	hypothetical protein	unknown function
OJRRIMKN_0135	95092	94865	-	CDS	hypothetical protein	unknown function
OJRRIMKN_0136	95421	95089	-	CDS	hypothetical protein	unknown function
OJRRIMKN_0137	95533	95396	-	CDS	hypothetical protein	unknown function
OJRRIMKN_0138	95826	95578	-	CDS	hypothetical protein	unknown function

OJRRIMKN_0139	96300	95863	-	CDS	Ndd-like nucleoid disruption protein	DNA, RNA and nucleotide metabolism
OJRRIMKN_0140	96649	96275	-	CDS	hypothetical protein	unknown function
OJRRIMKN_0141	97594	96695	-	CDS	RIIB lysis inhibitor	lysis
OJRRIMKN_0142	99855	97594	-	CDS	RIIA lysis inhibitor	lysis
OJRRIMKN_0143	100102	99866	-	CDS	hypothetical protein	unknown function
OJRRIMKN_0144	100660	100106	-	CDS	hypothetical protein	unknown function
OJRRIMKN_0145	102018	100732	-	CDS	DNA topoisomerase II	DNA, RNA and nucleotide metabolism
OJRRIMKN_0146	103916	102018	-	CDS	DNA topoisomerase II large subunit	DNA, RNA and nucleotide metabolism
OJRRIMKN_0147	104148	103984	-	CDS	FmdB-like transcriptional regulator	transcription regulation
OJRRIMKN_0148	104588	104148	-	CDS	unknown function	unknown function
OJRRIMKN_0149	105204	104611	-	CDS	hypothetical protein	unknown function
OJRRIMKN_0150	105469	105191	-	CDS	hypothetical protein	unknown function
OJRRIMKN_0151	106062	105466	-	CDS	hypothetical protein	unknown function
OJRRIMKN_0152	106431	106102	-	CDS	hypothetical protein	unknown function
OJRRIMKN_0153	106715	106491	-	CDS	hypothetical protein	unknown function
OJRRIMKN_0154	107052	106789	-	CDS	hypothetical protein	unknown function
OJRRIMKN_0155	107443	107141	-	CDS	hypothetical protein	unknown function
OJRRIMKN_0156	108162	107881	-	CDS	tail protein	tail
OJRRIMKN_0157	108830	108159	-	CDS	exonuclease	DNA, RNA and nucleotide metabolism
OJRRIMKN_0158	109111	108827	-	CDS	hypothetical protein	unknown function
OJRRIMKN_0159	109420	109112	-	CDS	hypothetical protein	unknown function
OJRRIMKN_0160	110773	109445	-	CDS	Dda-like helicase	DNA, RNA and nucleotide metabolism
OJRRIMKN_0161	111060	110770	-	CDS	hypothetical protein	unknown function
OJRRIMKN_0162	111778	111242	-	CDS	nucleoside triphosphate pyrophosphohydrolase	DNA, RNA and nucleotide metabolism
OJRRIMKN_0163	112811	111786	-	CDS	DNA primase	DNA, RNA and nucleotide metabolism
OJRRIMKN_0164	113075	112815	-	CDS	DksA-like zinc-finger protein	other
OJRRIMKN_0165	113349	113131	-	CDS	hypothetical protein	unknown function
OJRRIMKN_0166	113834	113346	-	CDS	hypothetical protein	unknown function
OJRRIMKN_0167	114571	113840	-	CDS	DNA methyltransferase	other
OJRRIMKN_0168	114944	114552	-	CDS	hypothetical protein	unknown function
OJRRIMKN_0169	115177	114941	-	CDS	hypothetical protein	unknown function
OJRRIMKN_0170	115524	115270	-	CDS	hypothetical protein	unknown function
OJRRIMKN_0171	115873	115604	-	CDS	hypothetical protein	unknown function
OJRRIMKN_0172	117353	115917	-	CDS	DnaB-like replicative helicase	DNA, RNA and nucleotide metabolism
OJRRIMKN_0173	117683	117363	-	CDS	head vertex assembly chaperone	head and packaging

OJRRIMKN_0174	118878	117721	-	CDS	DNA repair protein	DNA, RNA and nucleotide metabolism
OJRRIMKN_0175	121652	118953	-	CDS	DNA polymerase	DNA, RNA and nucleotide metabolism
OJRRIMKN_0176	122028	121699	-	CDS	hypothetical protein	unknown function
OJRRIMKN_0177	122312	122025	-	CDS	hypothetical protein	unknown function
OJRRIMKN_0178	122743	122381	-	CDS	translation repressor	other
OJRRIMKN_0179	123310	122747	-	CDS	clamp loader of DNA polymerase	DNA, RNA and nucleotide metabolism
OJRRIMKN_0180	124311	123313	-	CDS	clamp loader of DNA polymerase	DNA, RNA and nucleotide metabolism
OJRRIMKN_0181	125035	124370	-	CDS	DNA polymerase processivity factor	DNA, RNA and nucleotide metabolism
OJRRIMKN_0182	125331	125062	-	CDS	RpbA RNA polymerase binding protein	DNA, RNA and nucleotide metabolism
OJRRIMKN_0183	126358	125387	-	CDS	single strand DNA binding protein	DNA, RNA and nucleotide metabolism
OJRRIMKN_0184	127033	126374	-	CDS	DNA helicase loader	DNA, RNA and nucleotide metabolism
OJRRIMKN_0185	127275	127030	-	CDS	late promoter transcriptional regulator	transcription regulation
OJRRIMKN_0186	127542	127279	-	CDS	transcriptional regulator	transcription regulation
OJRRIMKN_0187	128487	127552	-	CDS	exonuclease	DNA, RNA and nucleotide metabolism
OJRRIMKN_0188	128792	129319	+	CDS	late sigma transcription factor	transcription regulation
OJRRIMKN_0189	129316	129531	+	CDS	hypothetical protein	unknown function
OJRRIMKN_0190	129524	129850	+	CDS	hypothetical protein	unknown function
OJRRIMKN_0191	129887	130933	+	CDS	SbcD-like subunit of palindrome specific endonuclease	DNA, RNA and nucleotide metabolism
OJRRIMKN_0192	130920	131207	+	CDS	hypothetical protein	unknown function
OJRRIMKN_0193	131191	132882	+	CDS	SbcC-like subunit of palindrome specific endonuclease	DNA, RNA and nucleotide metabolism
OJRRIMKN_0194	133588	132869	-	CDS	homing endonuclease	DNA, RNA and nucleotide metabolism
OJRRIMKN_0195	133645	133839	+	CDS	hypothetical protein	unknown function
OJRRIMKN_0196	133913	135622	+	CDS	hypothetical protein	unknown function
OJRRIMKN_0197	135682	135972	+	CDS	hypothetical protein	unknown function
OJRRIMKN_0198	135969	136199	+	CDS	hypothetical protein	unknown function
OJRRIMKN_0199	136196	136453	+	CDS	hypothetical protein	unknown function
OJRRIMKN_0200	136450	137028	+	CDS	dihydrofolate reductase	DNA, RNA and nucleotide metabolism
OJRRIMKN_0201	137030	137905	+	CDS	thymidylate synthase	DNA, RNA and nucleotide metabolism
OJRRIMKN_0202	137945	140197	+	CDS	ribonucleotide reductase large subunit	DNA, RNA and nucleotide metabolism
OJRRIMKN_0203	140234	141409	+	CDS	ribonucleoside diphosphate reductase small subunit	DNA, RNA and nucleotide metabolism
OJRRIMKN_0204	141372	141701	+	CDS	unknown function	unknown function
OJRRIMKN_0205	141705	142070	+	CDS	endonuclease	DNA, RNA and nucleotide metabolism
OJRRIMKN_0206	142051	143208	+	CDS	RNA ligase and tail fiber protein attachment catalyst	tail
OJRRIMKN_0207	143217	143528	+	CDS	Rz-like spanin	lysis
OJRRIMKN_0208	143525	143839	+	CDS	Rz-like spanin	lysis

OJRRIMKN_0209	143824	143997	+	CDS	hypothetical protein	unknown function
OJRRIMKN_0210	143994	144878	+	CDS	polynucleotide kinase	other
OJRRIMKN_0211	144931	145467	+	CDS	hypothetical protein	unknown function
OJRRIMKN_0212	145460	145744	+	CDS	hypothetical protein	unknown function
OJRRIMKN_0213	145686	146240	+	CDS	dCMP deaminase	DNA, RNA and nucleotide metabolism
OJRRIMKN_0214	146256	146597	+	CDS	head morphogenesis	head and packaging
OJRRIMKN_0215	146608	146859	+	CDS	hypothetical protein	unknown function
OJRRIMKN_0216	147174	147455	+	CDS	hypothetical protein	unknown function
OJRRIMKN_0217	147465	147887	+	CDS	hypothetical protein	unknown function
OJRRIMKN_0218	147929	148495	+	CDS	hypothetical protein	unknown function
OJRRIMKN_0219	148506	148868	+	CDS	hypothetical protein	unknown function
OJRRIMKN_0220	148868	149137	+	CDS	hypothetical protein	unknown function
OJRRIMKN_0221	149263	149556	+	CDS	hypothetical protein	unknown function
OJRRIMKN_0222	149559	149798	+	CDS	hypothetical protein	unknown function
OJRRIMKN_0223	149783	149953	+	CDS	hypothetical protein	unknown function
OJRRIMKN_0224	150257	150697	+	CDS	hypothetical protein	unknown function
OJRRIMKN_0225	150694	150957	+	CDS	hypothetical protein	unknown function
OJRRIMKN_0226	150959	151180	+	CDS	hypothetical protein	unknown function
OJRRIMKN_0227	151177	151791	+	CDS	unknown function	unknown function
OJRRIMKN_0228	151772	153301	+	CDS	ATP-dependent DNA ligase	DNA, RNA and nucleotide metabolism
OJRRIMKN_0229	153378	153947	+	CDS	hypothetical protein	unknown function
OJRRIMKN_0230	154036	154239	+	CDS	hypothetical protein	unknown function
OJRRIMKN_0231	154242	154418	+	CDS	hypothetical protein	unknown function
OJRRIMKN_0232	154415	154579	+	CDS	hypothetical protein	unknown function
OJRRIMKN_0233	154660	154950	+	CDS	hypothetical protein	unknown function
OJRRIMKN_0234	154961	155272	+	CDS	hypothetical protein	unknown function
OJRRIMKN_0235	155259	155636	+	CDS	hypothetical protein	unknown function
OJRRIMKN_0236	156742	155633	-	CDS	baseplate hub	tail
OJRRIMKN_0237	156812	157387	+	CDS	baseplate hub distal subunit	tail
OJRRIMKN_0238	157384	159174	+	CDS	baseplate hub	tail
OJRRIMKN_0239	159185	160258	+	CDS	baseplate tail tube cap	tail
OJRRIMKN_0240	160268	161131	+	CDS	tail tube	tail
OJRRIMKN_0241	161160	161903	+	CDS	baseplate hub	tail
OJRRIMKN_0242	161900	162454	+	CDS	baseplate hub	tail
OJRRIMKN_0243	162455	162847	+	CDS	baseplate wedge subunit	tail

OJRRIMKN_0244	163337	162882	-	CDS	hypothetical protein	unknown function
OJRRIMKN_0245	163569	163345	-	CDS	hypothetical protein	unknown function
OJRRIMKN_0246	163855	163571	-	CDS	hypothetical protein	unknown function
OJRRIMKN_0247	164211	163927	-	CDS	hypothetical protein	unknown function
OJRRIMKN_0248	164697	164227	-	CDS	hypothetical protein	unknown function
OJRRIMKN_0249	164852	164694	-	CDS	hypothetical protein	unknown function
OJRRIMKN_0250	165499	164849	-	CDS	hypothetical protein	unknown function
OJRRIMKN_0251	167754	165538	-	CDS	hypothetical protein	unknown function
OJRRIMKN_0252	168082	167762	-	CDS	hypothetical protein	unknown function
OJRRIMKN_0253	168597	168082	-	CDS	hypothetical protein	unknown function
OJRRIMKN_0254	168836	168597	-	CDS	hypothetical protein	unknown function
OJRRIMKN_0255	169110	168823	-	CDS	hypothetical protein	unknown function
OJRRIMKN_0256	169277	169110	-	CDS	hypothetical protein	unknown function
OJRRIMKN_0257	169595	169317	-	CDS	hypothetical protein	unknown function
OJRRIMKN_0258	170565	169654	-	CDS	DNA methyltransferase	other
OJRRIMKN_0259	170819	170562	-	CDS	hypothetical protein	unknown function
OJRRIMKN_0260	171064	170819	-	CDS	hypothetical protein	unknown function
OJRRIMKN_0261	172068	171061	-	CDS	nucleotidyltransferase	DNA, RNA and nucleotide metabolism
OJRRIMKN_0262	172445	172068	-	CDS	hypothetical protein	unknown function
OJRRIMKN_0263	172669	172442	-	CDS	hypothetical protein	unknown function
OJRRIMKN_0264	173270	172854	-	CDS	hypothetical protein	unknown function
OJRRIMKN_0265	173542	173273	-	CDS	hypothetical protein	unknown function
OJRRIMKN_0266	174669	173611	-	CDS	cytidyltransferase	other
OJRRIMKN_0267	175760	174846	-	CDS	lipoprotein	other
OJRRIMKN_0268	176095	175832	-	CDS	starvation-inducible transcriptional regulator	transcription regulation

Table S2. Number of CDSs assigned to functional categories using pharokka.

	phage KP1LMA	phage phiKp_26
**Description**	**Count**	**Count**
**CDS**	268	275
**connector**	3	3
**DNA, RNA and nucleotide metabolism**	42	41
**head and packaging**	13	14
**integration and excision**	0	0
**lysis**	7	7
**moron, auxiliary metabolic gene and host takeover**	4	4
**other**	14	14
**tail**	31	30
**transcription regulation**	6	5
**unknown function**	148	157
**tRNAs**	2	2
**CRISPRs**	0	0
**tmRNAs**	0	0
**VFDB_Virulence_Factors**	0	0
**CARD_AMR_Genes**	0	0

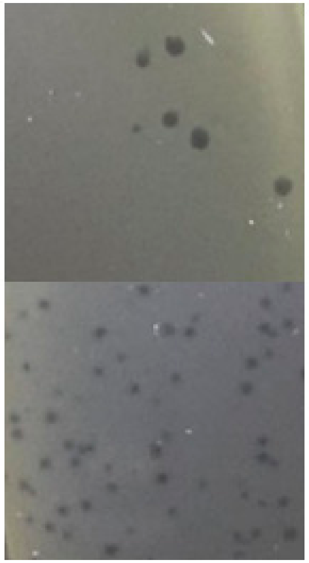

Figure S1. Plaques formed on *E. coli* ATCC 13706 (A) were completely clear whereas plaques formed on the original host (B) presented turbid aspect and were smaller.
